# Supplementary material for: A higher order PUF complex is central to regulation of C. elegans germline stem cells
Source: bioRxiv. 2024 Jun 14:2024.06.14.599074. Preprint. [Version 1] doi: 10.1101/2024.06.14.599074 (PMC11195197; doi:10.1101/2024.06.14.599074)
Supplement: Supplement 1 [file media-1.pdf]

Supplementary Information

Supplementary Table 1. Crystallographic data collection and refinement statistics.

	**FBF-2 RBD/FBEa***
Resolution range (Å)	20.0-2.29 (2.33-2.29)[1]
Space group	$P6_1$
Unit cell dimensions<br>a, b, c (Å)<br>α, β, γ (°)	99.0, 99.0, 107.2<br>90, 90, 120
Unique reflections[2]	26610 (1305)
Multiplicity	7.3 (7.4)
Completeness (%)	99.9 (99.9)
Mean I/sigma(I)	13.5 (2.0)
Wilson B-factor	50.7
R-meas	0.15 (0.80)
R-pim	0.06 (0.29)
**Refinement**	
Reflections used in refinement	26063
Reflections used for R-free	1963
R-work	0.191 (0.257)
R-free	0.232 (0.299)
Number of atoms	
protein	3157
RNA	212
solvent	89
RMSD bonds (Å)	0.002
RMSD angles (°)	0.371
Ramachandran favoured (%)	98.2
Ramachandran outliers (%)	0
Average B-factors (Å^2)	
protein	65.2
RNA	79.1
solvent	58.3

[1]The highest-resolution shell is shown in parentheses.

[2]Statistics for the highest-resolution shell are shown in parentheses.

Supplementary Table 2. Statistical significance for GLD-1 quantitation comparisons

Figure	Strains compared	Region (µm from distal end)	p-value	Sig[1]
	**Endogenous**			
3D	FBEa^{*m} (n=52)<br>vs control (n=40)	0-10<br>70-80<br>90-100	<0.001<br>0.644<br>0.751	***<br>ns<br>ns
3E[2]	FBEa^{m} (n=64)<br>vs control (n=37)	0-10<br>70-80<br>90-100	<0.001<br>0.365<br>0.416	***<br>ns<br>ns
3D, E	FBEa^{m} (n=64)<br>vs FBEa^{m} FBEa^{*m} (n=26)	0-10<br>70-80<br>90-100	0.663<br>0.001<br><0.001	ns<br>**<br>***
3F	FBEa^{m} FBEa^{*m} (n=26)<br>vs control (n=20)	0-10<br>70-80<br>90-100	<0.001<br>0.036<br>0.006	***<br>*<br>**
3G	FBEa^{*m} FBEb^{m} (n=49)<br>vs control (n=37)	0-10<br>70-80<br>90-100	0.018<br>0.025<br>0.009	*<br>*<br>**
3D, G[3]	FBEa^{*m} FBEb^{m}(n=49)<br>vs FBEa^{*m} (n=31)[2]	0-10<br>70-80<br>90-100	0.568<br>0.007<br>0.002	ns<br>**<br>**
	**Reporter**			
S3B	FBEa^{m} (n=24) vs wt (n=25)[2]	0-10<br>70-80<br>90-100	<0.001<br>0.693<br>0.350	***<br>ns<br>ns
S3B	FBEa^{m} FBEa^{*m} (n=19)<br>vs wt (n=25)	0-10<br>70-80<br>90-100	<0.001<br>0.010<br>0.003	***<br>*<br>**

[1] Significance: *** < 0.001; ** < 0.01; * < 0.05; ns > 0.05
[2] Endogenous FBEa data from Carrick et al, 2024. Reporter FBEa data from this work.
[3] Separate data set from Figure 3D, done in same experiment as FBEa^{*m} FBEb^{m}

Supplementary Table 3. Cryo-EM data collection and processing.

	1 FBF-2	2 FBF-2
EMDB code	EMD-45096	EMD-45097
Magnification	45,000	
Voltage (kV)	200	
Electron exposure ($e^-/Å^2$)	54	
Defocus range (μm)	-1.0 to -2.5	
Pixel size (Å)	0.932	
Symmetry imposed	C1	
Initial particle images (no.)	1,263,785	
Final particle images (no.)	252,126	110,843
Map resolution (Å) (FSC = 0.143)	4.4	6.4

Supplementary Table 4. Table of closely spaced adjacent FBF-2 binding sites identified in analysis of eCLIP data.

See enclosed Excel spreadsheet.

Supplementary Table 5. RNA sequences used in the EMSA RNA-binding experiments.

**RNA**	**Sequence (with 3´-Cy5)**[1]
FBEa-FBEa*	AUCA**UGU**GCCAUACAUCA**UGUU**GCCAUUU
FBEa^{m}-FBEa*	AUCAACAGCCAUACAUCA**UGUU**GCCAUUU
FBEa-FBEa*^{m}	AUCA**UGU**GCCAUACAUCAACA**U**GCCAUUU
FBEa^{m}-FBEa*^{m}	AUCAACAGCCAUACAUCAACA**U**GCCAUUU
FBEa*	AUCA**UGUU**GCCAUUU

[1]Mutations in red. FBE sequences underlined.

Supplementary Table 6. Sequences of guide RNAs and repair templates to create CRISPR alleles.

Description	Guide (5´-3´)	Repair template (5´-3´)
FBEa	aaaaatggcaacatgatgta	gttcgttctcaccattttaggttaccatagaatcaACAgcGatacatcatgttgccatttttccccctctcatctcccc
FBEa*	aaaaatggcaacatgatgta	gttcgttctcaccattttaggttaccatagaatcatgtgccatacatcaACAtgccatttttccccctctcatctcccc
FBEa-FBEa*	aaaaatggcaacatgatgta	gttcgttctcaccattttaggttaccatagaatcaACAgcGatacatcaACAtgccatttttccccctctcatctcccc
FBEb	ataacTGTgaaaaataaagg	cccattcatactacctcgaatgccaaagcacccccttttattttcACAgttatcttaacgctaacccctgtagaatcttcccggt

Supplementary Table 7. Strains used in this manuscript.

	**Strain name**	**allele**	**Comments**
N2			
sur-5	JK4864	*qIS147*	sur-5::GFP marked wt control
unc-119(ed3) III; teIs1 IV	TX189	*teIs1*	oma-1::GFP prevents GFP silencing
**Endogenous *gld-1* Crispr alleles**			**Oligos to detect FBE mutations (5´-3´)**
gld-1 FBEa^{m} [1]	JK6540	*q1242*	slc299 GAAGTACCCAACAACCACTTCG<br>prHJS401 TGGCAACATGATGTATCGCTGT
gld-1 FBEa*^{m}	JK6531	*q1234*	slc299 GAAGTACCCAACAACCACTTCG<br>slc301 GAGAGGGGGAAAAAATGGCATGT
gld-1 FBEa^{m}-FBEa*^{m}	JK6541	*q1243*	Use primer sets for FBEa and a*
gld-1 FBEa*^{m}-FBEb^{m}	JK6736	*q1297*	Use primer set for FBEa*<br>For FBEb:<br>slc299 GAAGTACCCAACAACCACTTCG<br>slc302 GGGTTAGCGTTAAGATAACTGT
**Reporter strains and Crispr alleles**			
rajSi50 FBEwt [2]	NIK50	*rajSi50*	slc314 GTCACCAAGTACACTTCCAGCAAG<br>slc301 GAGAGGGGGAAAAAATGGCATGT
rajSi50 FBEa^{m} [1]	JK6551	*q1274*	slc314 GTCACCAAGTACACTTCCAGCAAG<br>prHJS401 TGGCAACATGATGTATCGCTGT
rajSi50 FBEa^{m}-FBEa*^{m}	JK6639	*q1275*	Use primer sets for FBEa and a*

[1]Carrick et al. [2] Theil et al.

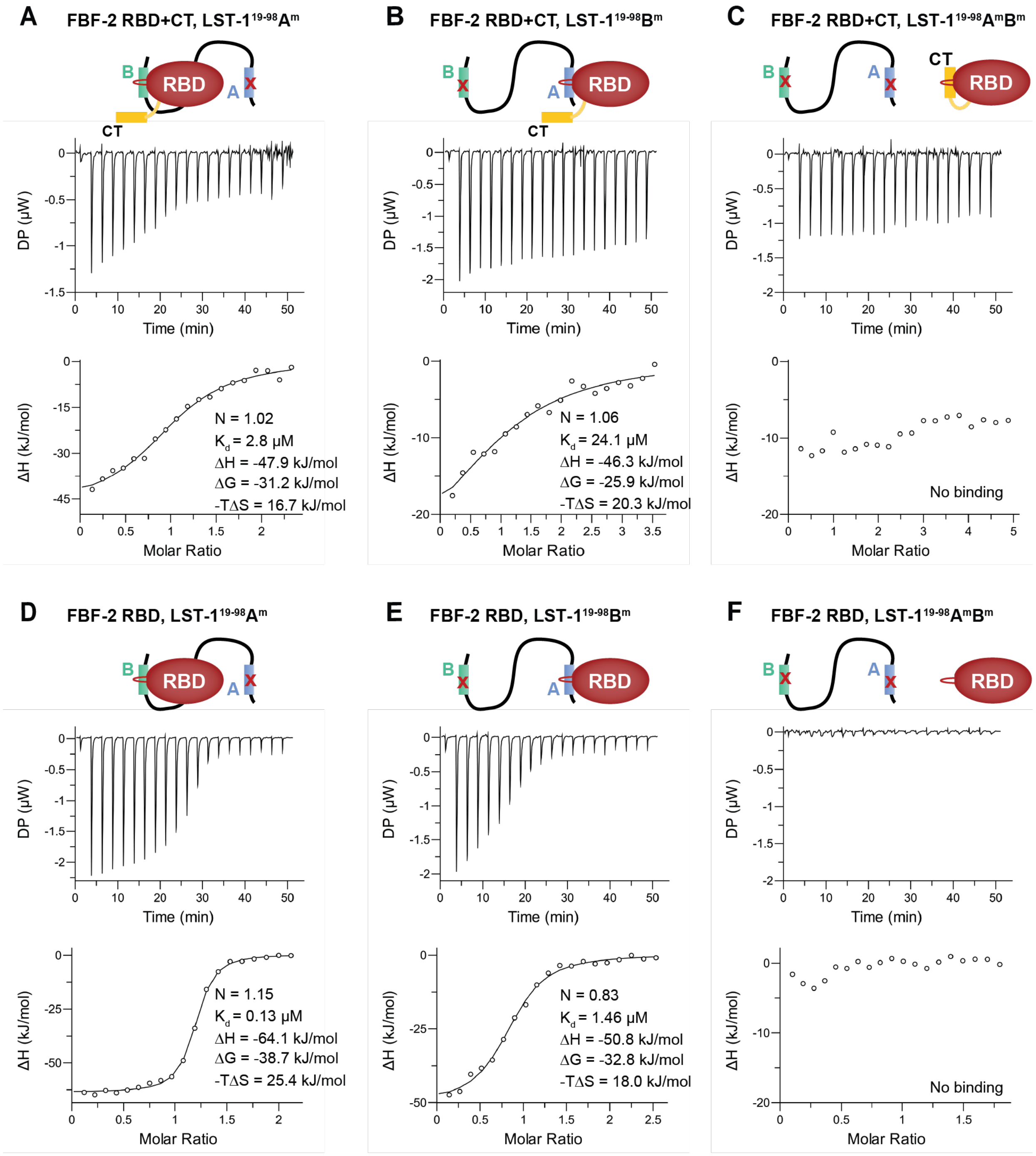

Supplementary Figure 1. LST-1 requires both PIMs for binding to two FBF-2 molecules. Representative ITC thermograms (top, differential power [DP] vs time) and corresponding titration curve-fitting graphs (bottom) for interaction of FBF-2 RBD+CT and (A) LST-$1^{19\text{-}98}$(A^m), (B) LST-$1^{19\text{-}98}$(B^m), and (C) LST-$1^{19\text{-}98}$ (A^mB^m). Representative ITC thermograms (top, DP vs

time) and corresponding titration curve-fitting graphs (bottom) for interaction of FBF-2 RBD and (D) LST-$1^{19\text{-}98}$(A^m), (E) LST-$1^{19\text{-}98}$(B^m), and (F) LST-$1^{19\text{-}98}$(A^mB^m). Thermodynamic parameters from one replicate indicated in bottom panels; thermodynamic parameters from two technical replicates are presented in **Table 1**. Experimental components indicated in diagrams above graphs.

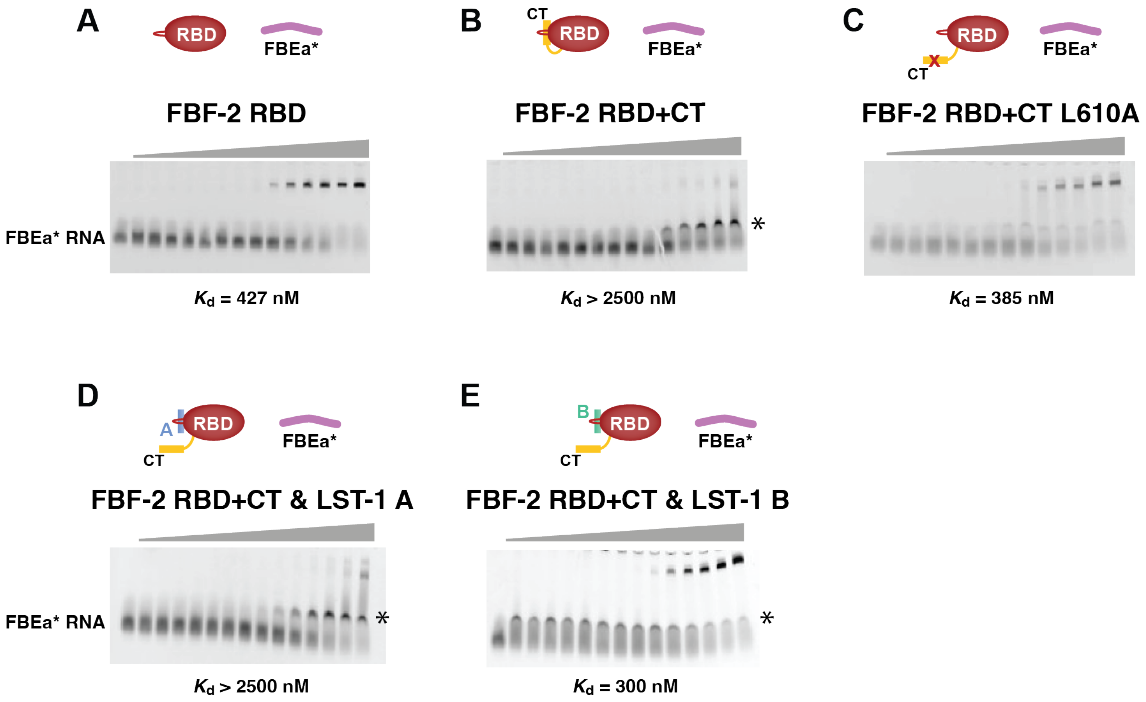

Supplementary Figure 2. FBF-2 binds to FBEa* RNA. Representative EMSA gels are shown for binding to FBEa* RNA (5´-AUCAUGUGCCAUAC-3´) by (A) FBF-2 RBD, (B) FBF-2 RBD+CT, (C) FBF-2 RBD+CT L610A, (D) FBF-2 RBD+CT with 150 μM LST-$1^{19\text{-}50}$ carrying PIM A, and (E) FBF-2 RBD+CT with 50 μM LST-$1^{67\text{-}98}$ carrying PIM B. Experimental components indicated in diagrams above gels. In panels B and D, we observed an intermediate band (*) that appears to be a non-specific interaction of FBF-2 RBD+CT, which was not observed for RBD. Similarly, LST-$1^{67\text{-}98}$ binds non-specifically to the RNA in panel E. We previously identified similar bands for non-specific binding to shorter RNAs[1]. We included these bands as part of the unbound RNA. Mean K_d values from at least three technical replicates are reported. See also **Figure 2F**.

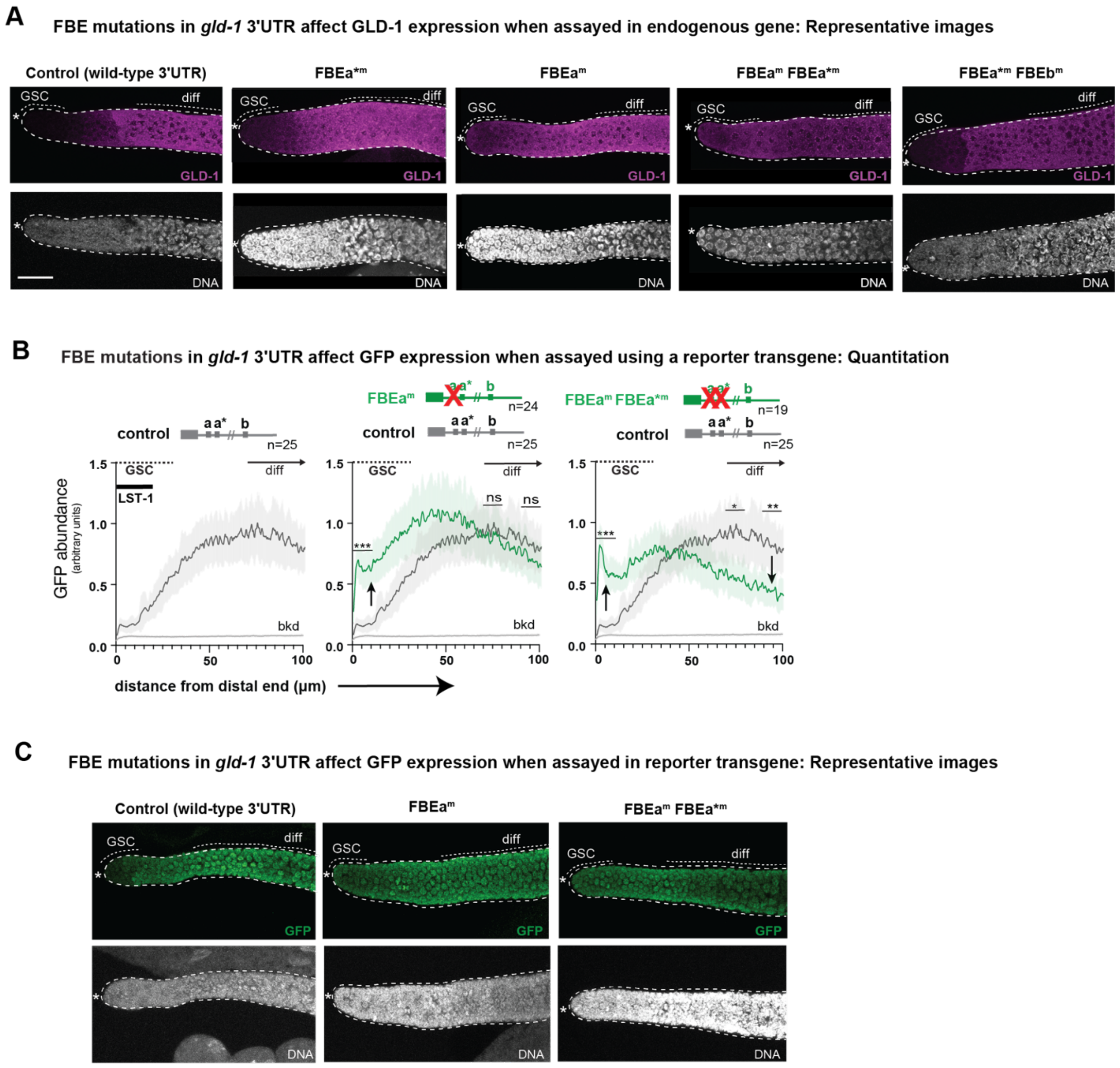

Supplementary Figure 3. Supplementary information for *gld-1* FBE mutants. (**A**) Representative z-projections of GLD-1 staining of FBE mutations generated in endogenous *gld-1*. Left to right: Thin dotted lines mark GSC pool and differentiation (diff); thick dotted line marks gonad boundary; asterisk marks distal end. Scale bar (bottom left), 20 µm for all panels in A and C. See Figure 3D-G for quantitation. See Carrick et al.[2] for images of GLD-1 in FBEa^{m} mutant (graph in Figure 3E). (**B**) ImageJ quantitation of GFP abundance expressed from a *gld-1* 3´UTR reporter transgene as a function of position in the distal gonad. Gray lines show GFP pattern in wild-type; green lines show GFP pattern in mutant. Shading is the 95% confidence interval.

Gonadal regions with GSCs (GSC) and differentiated (diff) germ cells are marked above; extent of LST-1 protein is marked with a thick black line in the control panel. P-values are given for pooled data in 0-10, 70-80 and 90-100 μm regions (black bars). P-values: *** $p < 0.001$, ** $p < 0.01$, * $p < 0.05$, ns (not significant) $p > 0.05$. See **Supplementary Table 2** for exact p-values. Left to right: Wild-type control (two replicates), FBEa^{m} (two replicates), and FBEa^{m} FBEa*^{m} double mutant (two replicates). Reporter constructs are shown above. Arrows indicate significant changes in GFP abundance relative to the wild-type control *gld-1* 3´UTR. Light grey line labeled “bkd” represents mean and 95% confidence interval for background staining in a wild-type animal not expressing the reporter construct (n=16, 1 replicate)[2]. (**C**) Representative z-projections of GFP reporter in extruded gonads with wild-type or mutated FBEs.

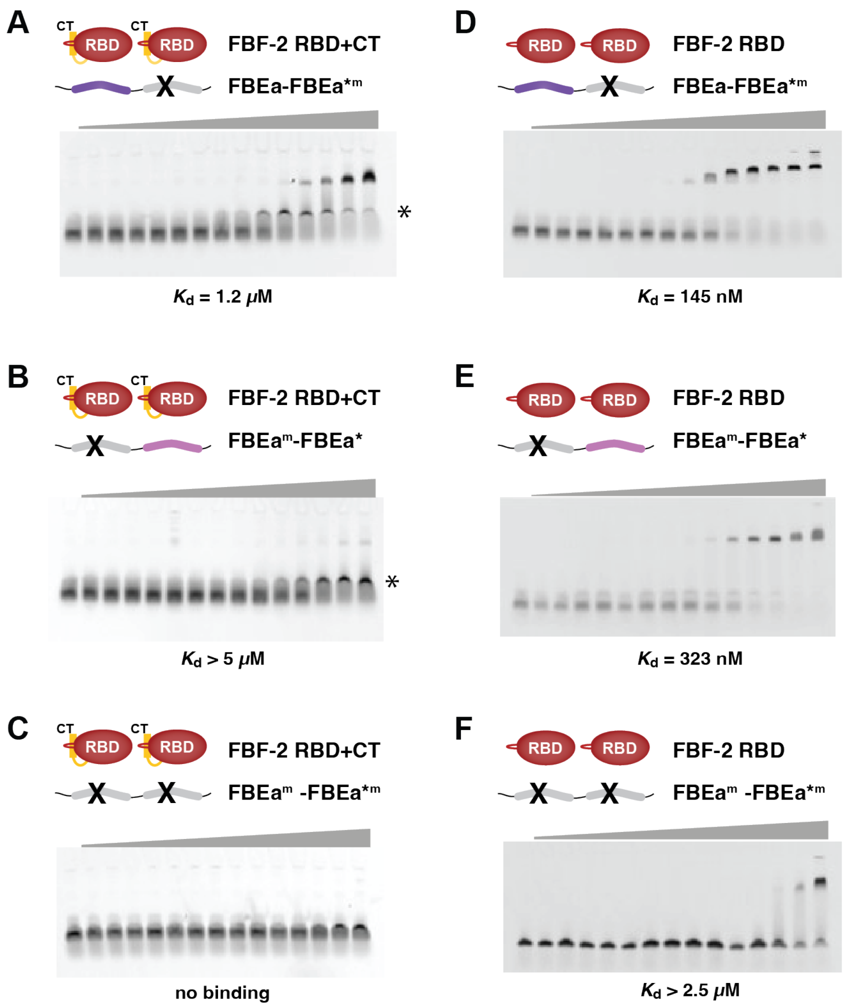

Supplementary Figure 4. Representative EMSA gels are shown for binding to FBEa-FBEa* RNA variants by (A-C) FBF-2 RBD+CT and (D-F) FBF-2 RBD. Experimental components indicated in diagrams above gels. LST-1 protein was not present. In panels A and B, we observed an intermediate band (*) that appears to be non-specific interaction of FBF-2 RBD+CT. We included these bands as part of the unbound RNA. Similar bands were detected previously with FBF-2 RBD+CT[1]. Mean K_d values from at least three technical replicates are reported. See also **Table 2**.

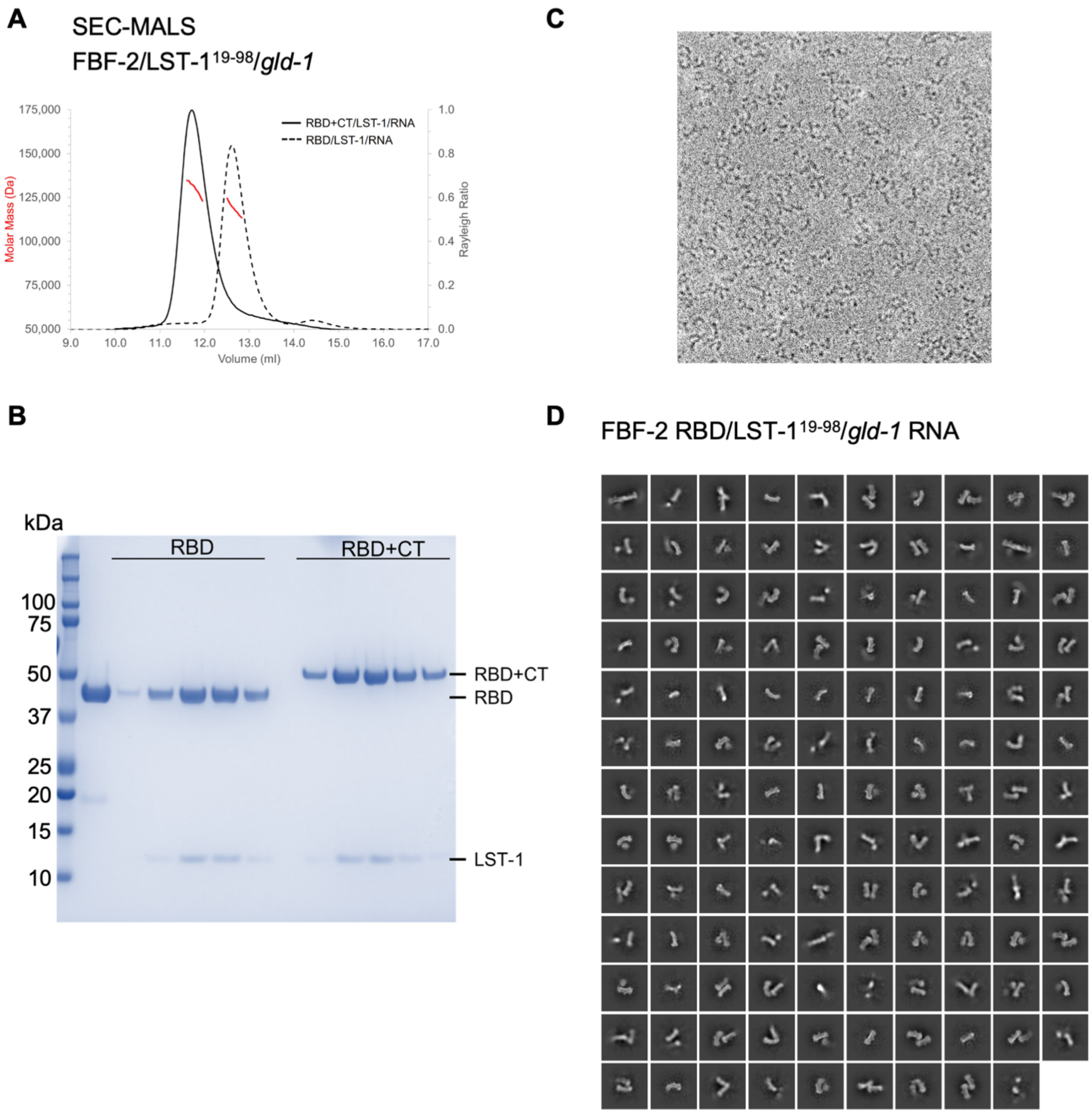

Supplementary Figure 5. Analyses of FBF-2/LST-1/ FBEa-FBEa* RNA complexes. (A) SEC-MALS analysis of FBF-2/LST-1$^{19\text{-}98}$/ FBEa-FBEa* RNA quaternary complexes. The peak for complexes with FBF-2 RBD+CT (solid black) had an apparent molecular mass of 130 kDa (red), which matches the calculated molecular weight of 125 kDa for a 2:1:1 complex of FBF-2 RBD+CT/LST-1$^{19\text{-}98}$/FBEa-FBEa*. The peak for complexes with FBF-2 RBD (dashed black line) had an apparent molecular mass of 119 kDa (red), which matches the calculated molecular weight of 119 kDa for a 2:1:1 complex of FBF-2/LST-1$^{19\text{-}98}$/FBEa-FBEa*. Molecular weights for individual components: FBF-2 RBD+CT, 53 kDa; FBF-2 RBD, 47 kDa; LST-1$^{19\text{-}98}$, 9.4 kDa, and

FBEa-FBEa* RNA, 8.6 kDa. (B) Coomassie-stained gel of SEC-MALS peak fractions. (C) Representative cryo-EM micrograph. (D) All 2D classes used for 3D reconstructions.

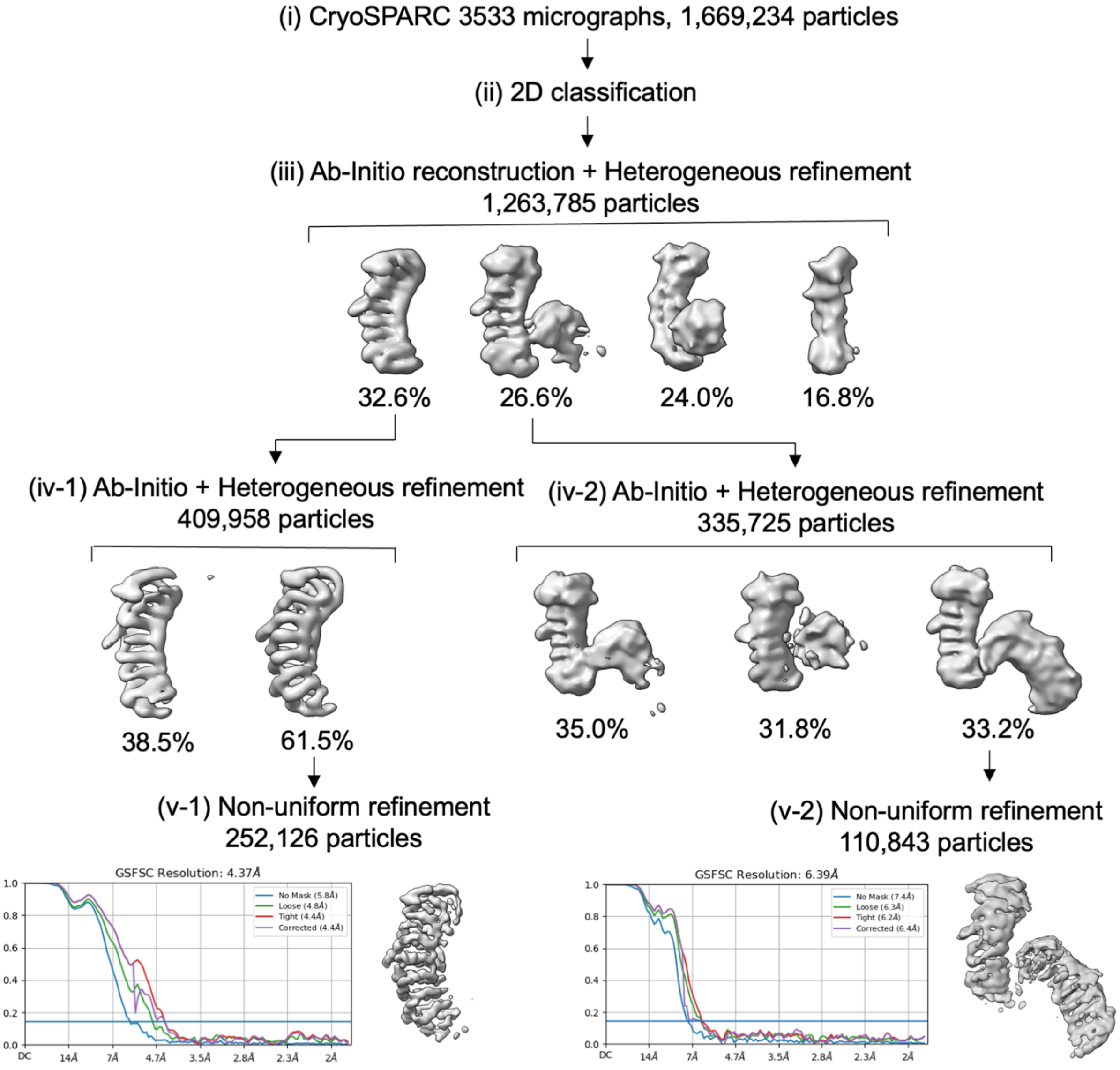

Supplementary Figure 6. Overview of cryo-EM processing scheme of FBF-2/LST-1/ FBEa-FBEa* RNA complex. Details are described in the Methods.

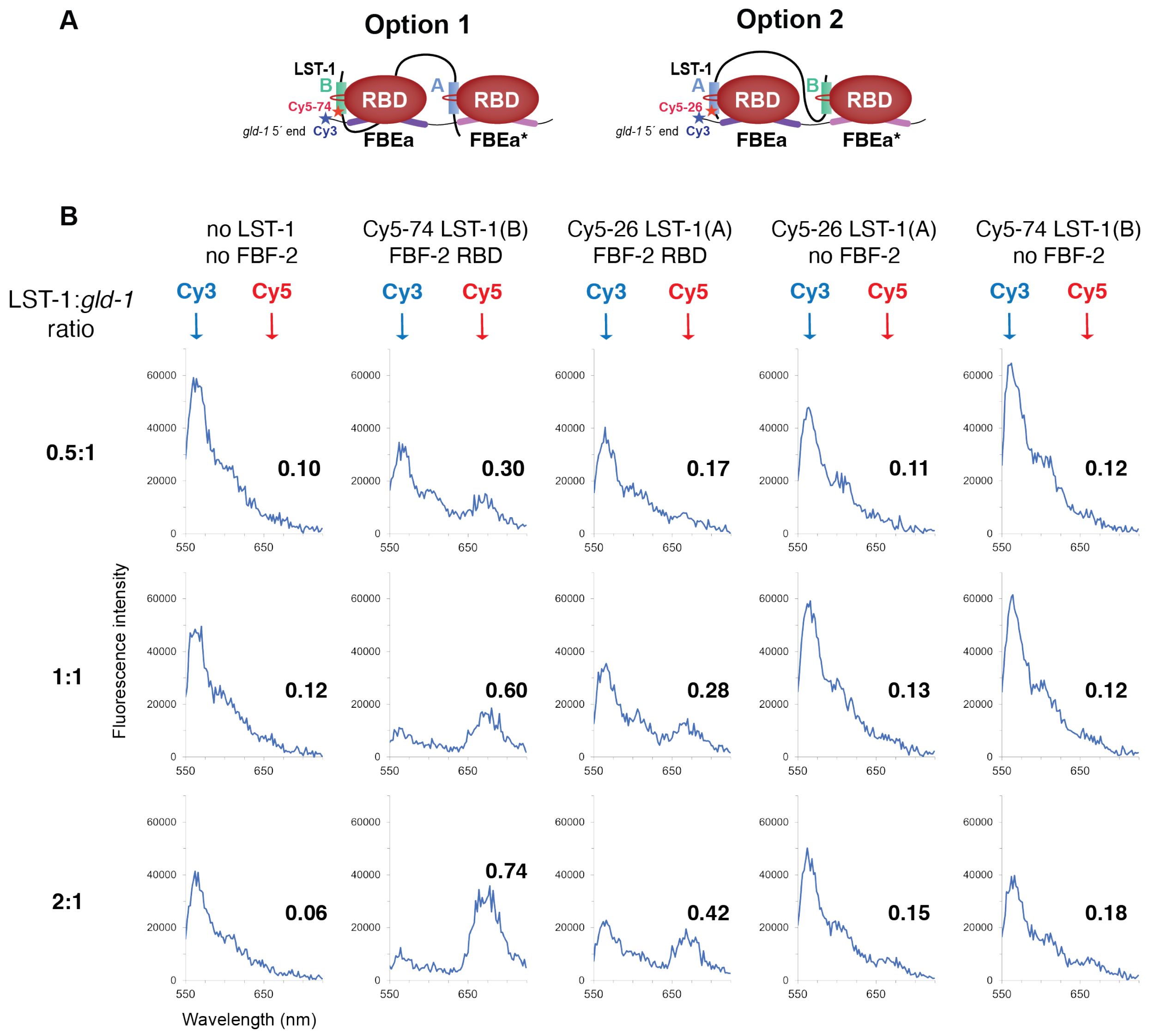

Supplementary Figure 7. FRET analysis of FBF-2/LST-1/ FBEa-FBEa* RNA complex. (A) Two possible orientations of LST-1 in the FBF-2/LST-1/ FBEa-FBEa* RNA complex. (B) Fluorescence spectra of excitation of Cy3-labeled RNA (blue arrow) and transfer to Cy5-labeled LST-1 (red arrow). In addition to complexes composed of Cy5-labeled LST-1, unlabeled FBF-2 RBD, and 5´-Cy3 labeled *gld-1* FBEa-FBEa* RNA, we also measured background levels of emission at 668 nm with samples of RNA only or RNA with LST-1 in the absence of FBF-2. FRET efficiencies were calculated as $I_{668}/(I_{668} + I_{564})$.

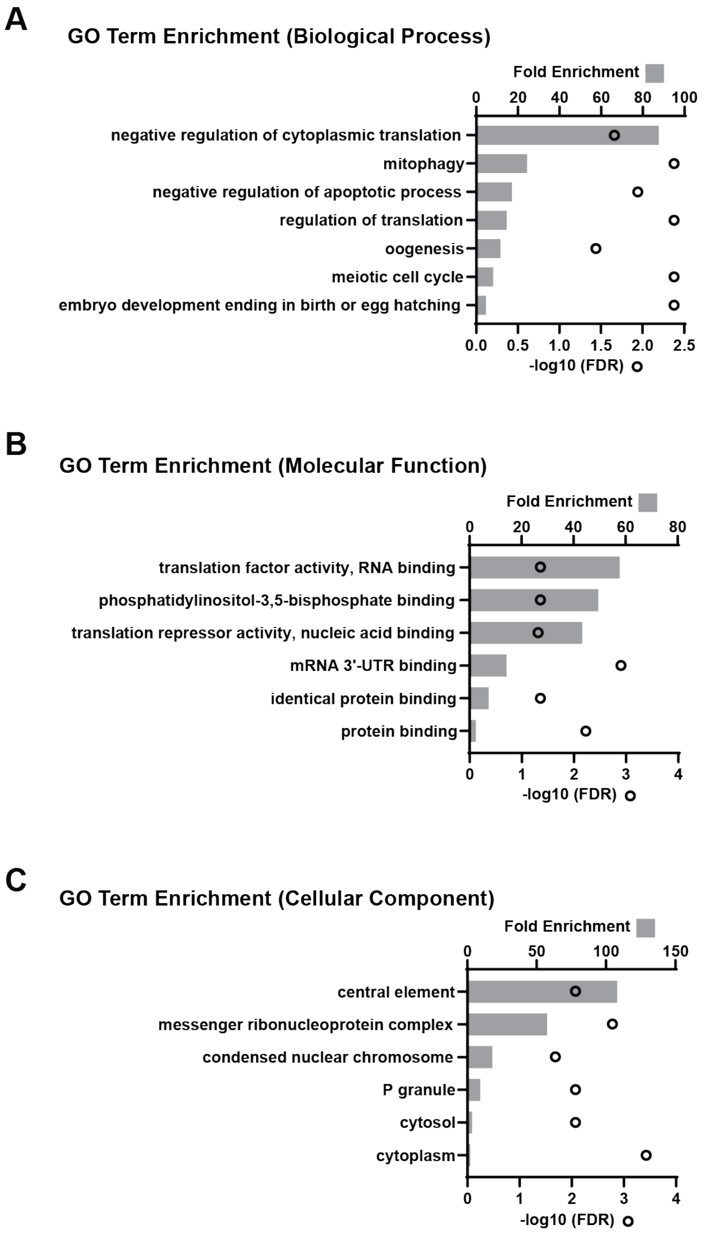

Supplementary Figure 8. GO term analysis of RNAs with adjacent FBEs. (A) Biological process GO term enrichment of genes that contain a peak with adjacent sites. Bars represent fold enrichment over background (top x-axis). Dots represent false discovery rate (FDR, bottom x-axis). Cutoffs for GO terms: fold enrichment ≥ 2 and FDR ≤ 0.05. (B) Molecular function GO term enrichment. Conventions and cutoffs as in (A). (C) Cellular component GO term enrichment. Conventions and cutoffs as in (A).

Supplementary References

1. Qiu C, Zhang Z, Wine RN, Campbell ZT, Zhang J, Hall TMT. Intra- and inter-molecular regulation by intrinsically-disordered regions governs PUF protein RNA binding. *Nat Commun* **14**, 7323 (2023).

2. Carrick BH*, et al.* PUF partner interactions at a conserved interface shape the RNA-binding landscape and cell fate in *Caenorhabditis elegans*. *Dev Cell* **59**, 661-675.e661-e667 (2024).
